# Remarkable impacts of probiotics supplementation in enhancing of the antioxidant status: results of an umbrella meta-analysis

**DOI:** 10.3389/fnut.2023.1117387

**Published:** 2023-08-11

**Authors:** Vali Musazadeh, Amir Hossein Faghfouri, Meysam Zarezadeh, Azin Pakmehr, Pooria Taghavi Moghaddam, Fateme Hamedi-Kalajahi, Arian Jahandideh, Zohreh Ghoreishi

**Affiliations:** ^1^Student Research Committee, Tabriz University of Medical Sciences, Tabriz, Iran; ^2^School of Nutrition and Food Sciences, Tabriz University of Medical Sciences, Tabriz, Iran; ^3^Maternal and Childhood Obesity Research Center, Urmia University of Medical Sciences, Urmia, Iran; ^4^Nutrition Research Center, Faculty of Nutrition and Food Science, Tabriz University of Medical Sciences, Tabriz, Iran; ^5^Faculty of Medicine, Tehran University of Medical Sciences, Tehran, Iran; ^6^Department of Pharmaceutics, School of Pharmacy, Ahvaz Jundishapur University of Medical Sciences, Ahvaz, Iran; ^7^Usern Office, Mazandaran University of Medical Sciences, Sari, Iran

**Keywords:** systematic review, probiotics, umbrella meta-analysis, malondialdehyde (MDA), oxidative stress biomarkers

## Abstract

**Introduction:**

Numerous meta-analyses have demonstrated the beneficial effects of probiotics on oxidative stress biomarkers, although some studies have contradictory results. Therefore, the current research was conducted to obtain a precise and definite understanding on the impact of probiotics on oxidative stress biomarkers in adults.

**Methods:**

We conducted a comprehensive systematic search of results on Scopus, PubMed, Embase, Web of Science, and Google Scholar dating up to March 2022. Fifteen meta-analyses were included in this umbrella meta-analysis. The random-effects model was employed to obtain the overall effect size. Subgroup analyses were carried out based on supplementation dosage and duration, mean age, and study population.

**Results:**

Our results indicated that probiotics supplementation meaningfully decreased serum malondialdehyde (MDA) (ES_WMD_ = −0.56, 95% CI: −0.72, −0.39; *p* < 0.001, and ES_SMD_ = −0.50, 95% CI: −0.66, −0.34; *p* < 0.001). Moreover, the findings showed that probiotics resulted in a significant increase in total antioxidant capacity (TAC) (ES_WMD_ = 29.18, 95% CI: 16.31, 42.04; *p* < 0.001, and ES_SMD_ = 0.25, 95% CI: 0.02, 0.47; *p* = 0.032), total glutathione (GSH) (ES_WMD_: 30.65; 95% CI: 16.94, 44.35, *p* < 0.001), and nitric oxide (NO) (ES_WMD_: 1.48; 95% CI: 0.31, 2.65, *p* = 0.013; I^2^ = 51.7%, *p* = 0.043).

**Discussion:**

Probiotics could be considered a strong agent in the reinforcement of antioxidant status and preventing the incidence of chronic diseases.

## 1. Introduction

The interaction of oxygen with certain molecules can cause the formation of highly reactive atoms named free radicals with unpaired electrons in their external shell, which can behave as oxidants (1). An imbalance between the reactive oxygen species (ROS), including superoxide (O2–), hydroxyl (OH–), and hydrogen peroxide (H2O2), and the body antioxidant defense system is defined as oxidative stress. Oxidative stress has been associated with a wide range of non-communicable and chronic diseases such as cardiovascular diseases, diabetes, Alzheimer's disease, cancer, and chronic obstructive pulmonary disease (2–4).

Intake of antioxidant vitamin supplementation, such as A, C, and E, as well as polyphenols and fruits and vegetables, is a common strategy to reinforce the antioxidant defense system (5–7). Nevertheless, evidence of a positive effect of probiotics in reducing oxidative stress and related diseases is also growing (5, 8). According to the World Health Organization (WHO) definition, probiotics are living microorganisms with certain benefits for human health when administered in a suitable amount (9). Certain strains of microorganisms can exhibit probiotic properties. For a strain to be called a “probiotic”, it must meet a number of requirements regarding safety, functionality, and technical suitability. The safety profile is determined based on strain origin, level of antibiotic resistance, and lack of association with pathogenic strains. Lactobacillus, Bifidobacterium, Lactococcus, Streptococcus, and Enterococcus species are the probiotic microorganisms mainly used in humans (10–14). More than 500 different bacteria reside in an adult human gastrointestinal tract as a source of probiotics, and many of the probiotic species used today have been isolated from the human gut. Besides the human GIT, dairy and dairy-related products, such as fermented milk and kefir, and non-dairy fermented substrates, such as meat and fruits, are good sources of probiotics (15–17).

Gut dysbiosis, which is the condition of the abnormal predominance of pathogenic over non-pathogenic microorganisms, is one of the confirmed causes of oxidative stress in the body (18, 19). Studies have demonstrated the beneficial role of probiotics in reconstruction of intestinal microbiota through various mechanisms including the maintenance of intestinal homeostasis (20).

Several studies have evaluated the effect of probiotic supplementation on a variety of disorders, and review studies have reported different results of probiotic effects. Several studies revealed no significant improvement in total antioxidant capacity (TAC) (21, 22) and malondialdehyde (MDA) after probiotic administration (23, 24), while in some other studies probiotics intake resulted in significant improvement in serum levels, TAC, total glutathione (GSH), and MDA (25, 26). Due to the reported contradictory results, we conducted the present umbrella meta-analysis to investigate the overall effect of probiotics on oxidative stress biomarkers in adults.

## 2. Material and methods

The Preferred Reporting Items for Systematic Reviews and Meta-Analyses (PRISMA) statement guidelines were used to develop the present umbrella meta-analysis. The protocol of this study has been registered in the international prospective register of systematic reviews (PROSPERO) under number CRD42023399865.

### 2.1. Search strategy and study selection

The international scientific databases of PubMed, Scopus, Web of Science, Cochrane Central Library, and EMBASE were searched for results dating up to March 2022. The following search pattern was utilized to explore related articles: “Probiotics” [Mesh] OR “probiotics” [All Fields] OR “probiotic” [All Fields] OR “Saccharomyces” [Title/Abstract] OR “Lactobacillus” [Title/Abstract] OR “Bifidobacterium” [Title/Abstract] OR “Lactobacillus casei” [Title/Abstract] OR “Bifidobacterium bifidum” [Title/Abstract] OR “Lactobacillus fermentum” [Mesh] OR “bifidobacterium” [All Fields] AND “Oxidative Stress” [MeSH terms] OR “Oxidative Stress” [Title/Abstract] OR “Total Antioxidant Capacity” [Title/Abstract] OR “antioxidant” [Title/Abstract] OR “Oxidant” [Title/Abstract] OR “reactive oxygen species” [Title/Abstract] OR “Malondialdehyde” [Title/Abstract] OR “glutathione” [Title/Abstract] OR “TAC” [Title/Abstract] OR “GSH” [Title/Abstract] OR “MDA” [Title/Abstract] OR “Nitric Oxide” [MeSH terms] AND “systematic review” [Publication Type] OR “meta-analysis” [Title/Abstract]. The wild-card term “^*^” was utilized to boost the sensitivity of the search method. The articles were limited to those written in the English language.

### 2.2. Inclusion and exclusion criteria

The PICO criteria for the present umbrella meta-analysis were as follows: Population/Patients (P): adults, 18 years old or above; Intervention (I): probiotics; Comparison (C): control or placebo group; Outcome (O): stress oxidative biomarkers including nitric oxide (NO), GSH, TAC, and MDA. Meta-analysis studies investigating the impact of probiotics supplementation on stress oxidative biomarkers and providing effect sizes and corresponding confidence intervals (CI) for each outcome were included in the present study. We excluded *in vitro* and *in vivo* studies, observational studies, case reports, quasi-experimental studies, and controlled clinical trials.

### 2.3. Quality assessment

Two independent reviewers (VM and FHK) examined the methodological quality of the included studies by the AMSTAR questionnaire (27). The AMSTAR questionnaire consists of 11 questions, and 11 is the maximum possible score. Articles with a score of 7 or higher were considered of good quality.

### 2.4. Data extraction

The screening and inclusion process of the studies based on the eligibility criteria were conducted by VM and FMK as two independent reviewers. We reviewed the abstracts and titles of the studies in the first step. Then, the full text of included studies was evaluated to determine the eligibility of the studies. Any disagreements were resolved by the senior author's decision (MZ). The name of the first author, year of the publication, sample size, intervention duration, study location, probiotics dosage, effect size [weighted mean difference (WMD) and standardized mean difference (SMD)], and confidence intervals (CIs) for NO, MDA, TAC, and GSH were extracted from the selected articles.

### 2.5. Data synthesis and statistical analysis

We calculated the overall effect size by pooling the effect size and CI of each included study. The analysis was performed separately for SMD and WMD due to their natural differences. The analysis was performed using the random-effects model in case of a high-heterogeneity amount, and the fixed-effects model was employed in case that the amount of heterogeneity was low. The I^2^ statistic and Cochran's Q test were used to determine between-study heterogeneity. Significant heterogeneity was considered as follows: I^2^ value >50% or *P* < 0.1 for the Q test. To recognize the probable sources of heterogeneity, subgroup analysis was performed according to the dose of probiotics, study population, sample size, and duration of intervention. We conducted a sensitivity analysis to determine whether the overall effect size was dependent on a particular study. The publication bias was evaluated using the Begg's and Egger's tests and funnel plot evaluation. In case of the presence of publication bias, trim-and-fill analysis was used to modify the publication bias. Egger's test and funnel plot evaluation were not performed when the total number of observations for each outcome was <10. All of the statistical analyses were performed using Stata software version 16.0 (Stata Corporation, College Station, TX, US). A *p*-value lower than 0.05 was considered significant.

## 3. Results

### 3.1. Selected studies and systematic reviews

**Figure 1:**
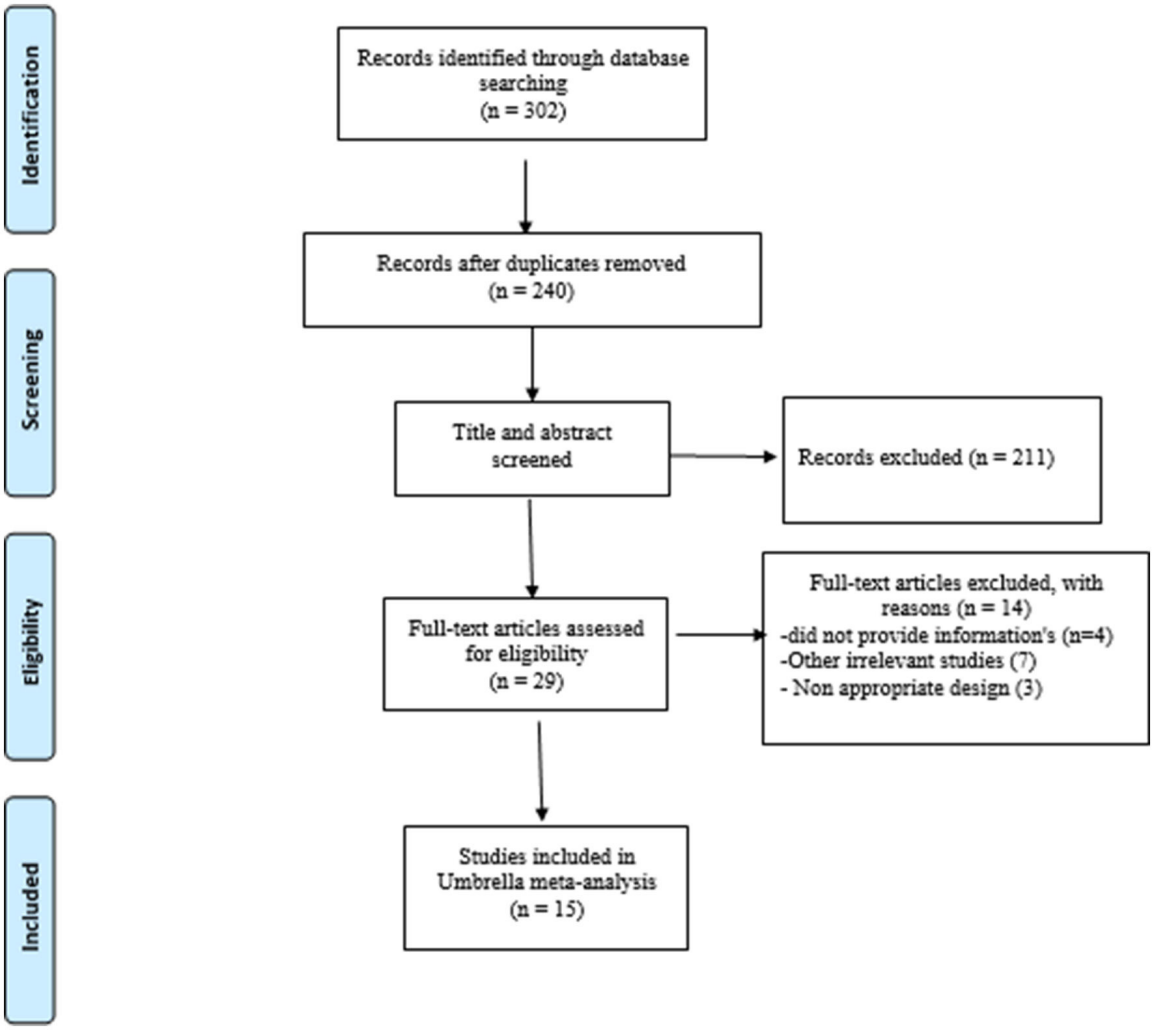
Flow chart of study selection.

A total number of 302 articles were obtained from the systematic search, among which 240 were thoroughly reviewed by titles and abstracts after 62 duplicate articles were excluded. Eventually, 29 articles were chosen for full-text examination, among which 15 meta-analyses were included in the umbrella meta-analysis. The PRISMA flowchart (Figure 1) represents the study selection process. The study participants' age ranged from 29 to 79, and the included studies were conducted between 2018 and 2021. The duration of interventions ranged from 6 to 14 weeks. The average dosage of probiotics in the current study varied from 1 × 10^10^ to 8 × 10^10^ CFU.

The studies were performed in Iran (23, 25, 26, 28–30), China (21, 22, 31–34), Malaysia (24), Egypt (35), and Brazil (36). The quality of trials included in the meta-analyses was assessed by the Cochrane Risk of Bias Tool (37) and Jadad scores (38), and almost all RCTs included in the meta-analyses were of high quality (Table 1). The characteristics of the studies qualified for this umbrella meta-analysis and the quality assessment results for the RCTs qualified in the meta-analyses are outlined in Table 1.

**Table 1:** Study characteristics of included studies.

**References**	**No. of studies in meta-analysis**	**Location Duration**	**No. of participants in meta-analysis**	**Age (year)**	**Intervention**	**Quality assessment scale and outcome**	**Outcomes**
Rudbane et al. (23)	2	Iran 8	106	49	*Lactobacillus, Bifidobacterium* 1 × 10^9^	Yes (Cochrane) 2/2 high	TAC↔ MDA↔
Ardeshirlarijani et al. (25)	13	Iran 8.5	809	56	*Lactobacillus, Bifidobacterium, Streptococcus* NR	Yes (Jadad) 13/13 high	TAC, GSH↑ MDA↓ NO↔
Hasain et al. (24)	4	Malysia 8wk	221	29	*Lactobacillus, Bifidobacterium, Streptococcus* 4 × 10^9^	Yes (Cochrane) 4/4 high	TAC, GSH↔ MDA↓ NO↑
Zamani et al. (30)	11	Iran 9wk	577	44	*Lactobacillus, Bifidobacterium, Streptococcus* 2.56 × 10^9^	Yes (Cochrane) 11/11 high	TAC↑ MDA↓ GSH↔
Krüger et al. (36)	3	Brazil 12wk	187	76	*Lactobacillus, Bifidobacterium* 2.3 × 10^9^	Yes (Cochrane) 1/3 high	MDA↓
Amirani et al. (28)	5	Iran 11wk	261	63	*Lactobacillus, Bifidobacterium* 4.5 × 10^9^	Yes (Cochrane) NR	MDA↓ TAC, GSH, NO ↔
Zhang et al. (31)	3	China 6wk	168	30	*Lactobacillus, Bifidobacterium, Streptococcus* 8 × 10^9^	Yes (Cochrane) 3/3 high	MDA↓ TAC, GSH, NO↑
Chan et al. (22)	6	China NR	411	29	NR 2 × 10^9^	Yes (Cochrane) 6/6 high	MDA↓ GSH ↑ TAC, NO ↔
Jiang et al. (33)	2	China 14wk	108	34	*Lactobacillus, Bifidobacterium, Streptococcus* 2 × 10^9^	Yes (Cochrane) 2/2 high	MDA↓ GSH ↔ TAC, NO↑
Den et al. (21)	3	China 12wk	198	79	*Lactobacillus, Bifidobacterium* 5.5 × 10^9^	Yes (Cochrane) 3/3 high	MDA↓ TAC, GSH, NO ↔
Chen et al. (32)	3	China 7wk	173	29	*Lactobacillus, Bifidobacterium* 2.5 × 10^9^	Yes (Jodad) 3/3 high	GSH↔ MDA↓ NO↑
Abdelqadir et al. (35)	3	Egypt 12wk	180	59	*Lactobacillus, Bifidobacterium* 4.05 × 10^9^	Yes (Cochrane) 3/3 high	TAC↑ MDA↓ GSH, NO↔
Bohlouli et al. (26)	4	Iran 11wk	320	57	*Lactobacillus, Bifidobacterium* NR	Yes (Cochrane) 1/4 high	TAC, GSH↑ MDA↓ NO↔
Wang et al. (34)	4	China 11wk	220	59	*Lactobacillus, Bifidobacterium* 2.53 × 10^9^	Yes (Cochrane) 4/4 high	TAC, GSH↑ MDA↓ NO↔
Tamtaji et al. (29)	7	Iran 12wk	331	63	*Lactobacillus, Bifidobacterium, Streptococcus* 3 × 10^9^	Yes (Cochrane) NR	MDA↓ TAC, GSH, NO ↔

NR, Not reported; TAC, total antioxidant capacity; GSH, total glutathione; MDA, malonaldehyde; NO, nitric oxide.

### 3.2. Methodological quality

A total of 6 of the 21 meta-analyses were rated as high quality, 13 as moderate quality, and 2 as low quality. Detailed results are presented in Table 2.

**Table 2:** Results of assessment of the methodological quality of meta-analysis.

	**Q1**	**Q2**	**Q3**	**Q4**	**Q5**	**Q6**	**Q7**	**Q8**	**Q9**	**Q10**	**Q11**	**Q12**	**Q13**	**Q14**	**Q15**	**Q16**	**Quality assessment**
Rudbane et al. (23)	No	Partial Yes	Yes	Partial Yes	Yes	Yes	Partial Yes	Yes	No	No	No	No	No	No	No	Yes	Low
Ardeshirlarijani et al. (25)	No	Yes	Yes	Partial Yes	Yes	Yes	Partial Yes	Yes	Yes	Yes	Yes	Yes	Yes	No	Yes	Yes	High
Chan et al. (22)	No	Yes	Yes	Partial Yes	No	Yes	Partial Yes	Yes	Yes	No	Yes	Yes	No	No	Yes	Yes	Moderate
Den et al. (21)	No	Partial Yes	Yes	Partial Yes	No	Yes	No	Yes	Yes	No	Yes	Yes	No	No	Yes	No	Low
Hasain et al. (24)	No	Partial Yes	Yes	Partial Yes	Yes	Yes	Partial Yes	Yes	Yes	Yes	Yes	Yes	Yes	No	No	Yes	Moderate
Zamani et al. (30)	No	Partial Yes	Yes	Partial Yes	Yes	Yes	Yes	Yes	Yes	Yes	Yes	Yes	Yes	Yes	Yes	Yes	High
Jiang et al. (33)	Yes	Partial Yes	Yes	Partial Yes	Yes	Yes	Yes	Yes	Yes	Yes	Yes	Yes	Yes	No	Yes	Yes	High
Krüger et al. (36)	Yes	Partial Yes	Yes	Partial Yes	Yes	Yes	No	Yes	Yes	No	Yes	Yes	Yes	Yes	No	Yes	Moderate
Amirani et al. (28)	No	Partial Yes	Yes	Partial Yes	Yes	Yes	Yes	Yes	Yes	No	Yes	Yes	No	No	No	Yes	Moderate
Zhang et al. (31)	No	Partial Yes	Yes	Yes	Yes	Yes	Partial Yes	Yes	Yes	Yes	Yes	Yes	Yes	Yes	Yes	Yes	High
Chen et al. (32)	No	Partial Yes	Yes	Partial Yes	Yes	Yes	Yes	Yes	Yes	No	Yes	Yes	Yes	Yes	No	Yes	Moderate
Abdelqadir et al. (35)	No	Yes	Yes	Partial Yes	Yes	Yes	No	Partial Yes	Yes	No	No	Yes	Yes	No	No	Yes	Moderate
Bohlouli et al. (26)	No	Yes	Yes	Partial Yes	Yes	Yes	No	Partial Yes	Yes	No	Yes	Yes	Yes	Yes	Yes	Yes	Moderate
Wang et al. (34)	Yes	Yes	No	Partial Yes	Yes	Yes	No	Partial Yes	Yes	No	Yes	Yes	Yes	Yes	Yes	Yes	Moderate
Tamtaji et al. (29)	No	Yes	Yes	Partial Yes	Yes	Yes	Yes	Partial Yes	Yes	No	Yes	Yes	Yes	Yes	Yes	Yes	High

1. Did the research questions and inclusion criteria for the review include the components of PICO? 2. Did the report of the review contain an explicit statement that the review methods were established prior to the conduct of the review and did the report justify any significant deviations from the protocol? 3. Did the review authors explain their selection of the study designs for inclusion in the review? 4. Did the review authors use a comprehensive literature search strategy? 5. Did the review authors perform study selection in duplicate? 6. Did the review authors perform data extraction in duplicate? 7. Did the review authors provide a list of excluded studies and justify the exclusions? 8. Did the review authors describe the included studies in adequate detail? 9. Did the review authors use a satisfactory technique for assessing the risk of bias (RoB) in individual studies that were included in the review? 10. Did the review authors report on the sources of funding for the studies included in the review? 11. If meta-analysis was performed, did the review authors use appropriate methods for statistical combination of results? 12. If meta-analysis was performed, did the review authors assess the potential impact of RoB in individual studies on the results of the meta-analysis or other evidence synthesis? 13. Did the review authors account for RoB in individual studies when interpreting/discussing the results of the review? 14. Did the review authors provide a satisfactory explanation for, and discussion of, any heterogeneity observed in the results of the review? 15. If they performed quantitative synthesis, did the review authors carry out an adequate investigation of publication bias (small study bias) and discuss its likely impact on the results of the review? 16. Did the review authors report any potential sources of conflict of interest, including any funding they received for conducting the review? Each question was answered with “Yes”, “Partial Yes” or “No”. When no meta-analysis was done, question 11, 12, and 15 were answered with “No meta-analysis conducted.”

### 3.3. The effects of probiotics supplementation on MDA based on WMD analysis

**Figure 2:**
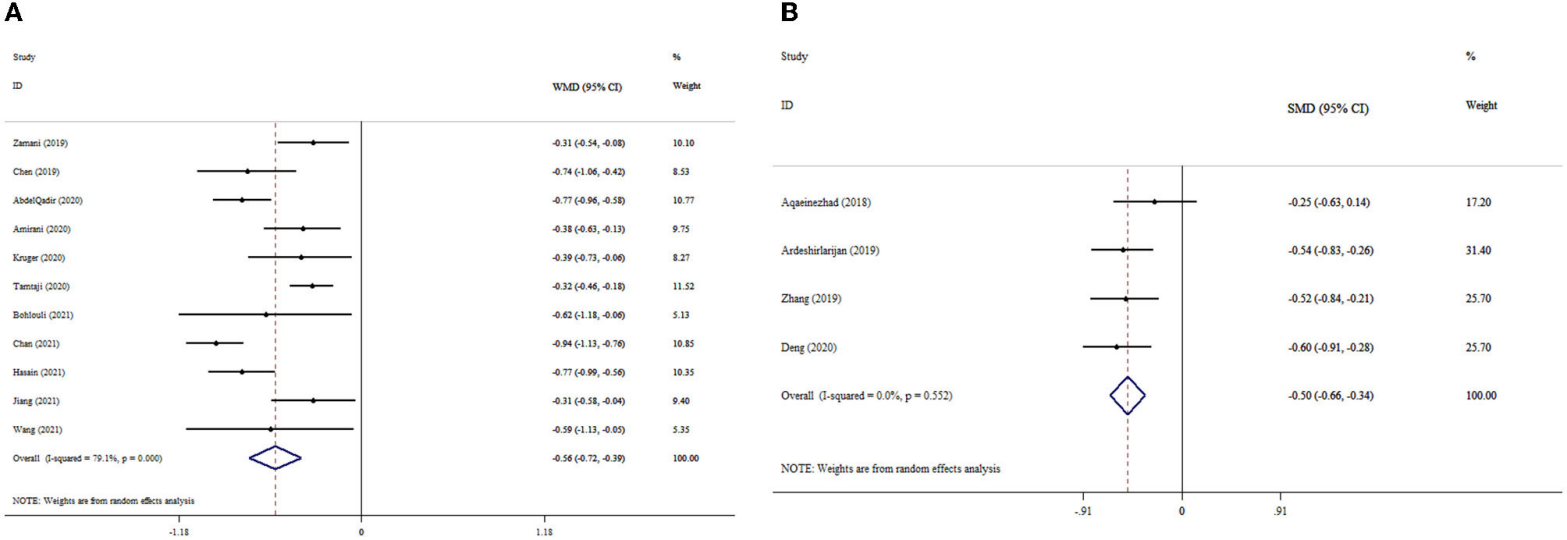
Forest plot with mean difference and 95% confidence intervals (CIs), and the effects of probiotics supplementation on MDA levels according to WMD **(A)** and SMD **(B)** analysis.

Eleven meta-analyses that included 2,605 participants revealed significant reduction in MDA levels (ES _WMD_ = −0.56, 95% CI: −0.72, −0.39; *p* < 0.001) (Figure 2A). Inter-study heterogeneity was found to be significant (I^2^ = 791%; *p* < 0.001). The subgroup analysis revealed that probiotic supplementation among subjects under 50 years of age with T2DM and a dosage of <0.4 × 10^10^ CFU substantially reduced MDA levels (Table 3). According to the sensitivity analysis, the removal of any of the studies did not affect the overall effect size estimate. Small-study effect was not detected using Egger's and Begg's tests (*p* = 0.896 and 0.999, respectively). Publication bias was also not identified through visual assessment of the funnel plot (Supplementary Figure S1).

**Table 3:** Pooled estimates of probiotics on stress oxidative biomarkers.

**Group**	**No. of comparisons**	**WMD (95% CI)**	***P*-value**	***I^2^* (%)**	***P*-heterogeneity**
**Probiotics supplementation on MDA levels**					
**Total**	11	−0.56 (−0.72, −0.39)	< 0.001	79.1	< 0.001
**Health condition**					
T2DM	6	−0.81 (−0.91, −0.71)	< 0.001	0.0	0.629
other	5	−0.33 (−0.43, −0.24)	< 0.001	0.0	0.987
**Age (years)**					
< 50	6	−0.49 (−0.69, - 0.30)	< 0.001	67.0	0.010
≥50	5	−0.62 (−0.89, −0.35)	< 0.001	84.2	< 0.001
**Duration (week)**					
< 10	3	−0.60 (−0.91, −0.29)	< 0.001	78.3	0.010
≥10	7	−0.46 (−0.63, −0.29)	< 0.001	63.1	0.012
NR	1	−0.94 (-1.13, −0.75)	< 0.001	-	-
**Dose (CFU)**					
< 0.4 × 10^10^	5	−0.57(−0.79, −0.35)	< 0.001	72.5	0.006
≥0.4 × 10^10^	6	−0.55 (−0.81, −0.28)	< 0.001	84.6	< 0.001
**Probiotics supplementation on TAC levels**					
**Total**	8	29.18 (16.31, 42.04)	< 0.001	13.01	0.328
**Health condition**					
T2DM	4	36.78 (19.33, 54.23)	< 0.001	13.0	0.327
other	4	19.70 (3.29, 36.12)	0.019	0.0	0.433
**Age (years)**					
< 50	3	46.94 (-2.39, 96.28)	0.062	50.2	0.134
≥50	5	30.00 (15.77, 44.22)	< 0.001	0.0	0.427
**Dose (CFU)**					
< 0.4 × 10^10^	5	41.36 (17.85, 64.88)	< 0.001	18.8	0.295
≥0.4 × 10^10^	3	22.22 (7.49, 36.94)	0.003	0.0	0.428
**Probiotics supplementation on GSH levels**					
**Total**	10	30.65 (16.94, 44.35)	< 0.001	24.5	0.218
**Health condition**					
T2DM	6	37.56 (15.24, 59.88)	< 0.001	43.7	0.114
other	4	22.32 (6.58, 38.05)	0.005	0.0	0.620
**Age (years)**					
< 50	5	23.82 (9.24, 38.40)	< 0.001	0.0	0.415
≥50	5	41.51 (17.90, 65.12)	< 0.001	42.6	0.137
**Duration (week)**					
< 10	2	8.08 (-35.21, 51.37)	0.715	0.0	0.647
≥10	7	31.80 (14.12, 49.47)	< 0.001	37.5	0.143
NR	1	44.02 (16.55, 71.49)	0.002	-	-
**Dose (CFU)**					
< 0.4 × 10^10^	5	37.18 (14.54, 59.81)	< 0.001	23.2	0.267
≥0.4 × 10^10^	5	25.40 (8.66, 42.14)	0.003	21.1	0.280
**Probiotics supplementation on NO**					
**Total**	8	1.48 (0.31, 2.65)	0.013	51.7	0.043
**Health condition**					
T2DM	5	1.87 (0.91, 2.84)	< 0.001	0.0	0.588
Other	3	1.51 (-1.55, 4.57)	0.334	75.1	0.018
**Age (years)**					
< 50	4	2.62 (1.40, 3.83)	< 0.001	12.1	0.332
≥50	4	0.13 (−0.95, 1.21)	0.815	0.0	0.698
**Duration (week)**					
< 10	2	2.57 (1.23, 3.90)	< 0.001	0.0	0.752
≥10	5	0.85 (−0.77, 2.48)	0.303	50.8	0.087
NR	1	1.79 (−0.39, 3.96)	0.107	-	-
**Dose (CFU)**					
< 0.4 × 10^10^	5	2.04 (0.60, 3.48)	0.005	29.8	0.223
≥0.4 × 10^10^	3	0.78 (-1.16, 2.73)	0.430	70.4	0.034

N, Number; NR, not reported.

### 3.4. The effects of probiotics supplementation on MDA based on SMD analysis

Our analysis findings based on four meta-analyses revealed that probiotic supplementation considerably decreased MDA levels (ES_SMD_= −0.50, 95% CI: −0.66, −0.34; *p* < 0.001), with no considerable between-studies heterogeneity (I^2^ = 0.0%; *p* = 0.552) (Figure 2B). The overall effect size was not affected by removing any study following the sensitivity analysis. Begg's test revealed no evidence of publication bias (*p* = 0.471).

### 3.5. The effects of probiotics supplementation on TAC based on WMD analysis

**Figure 3:**
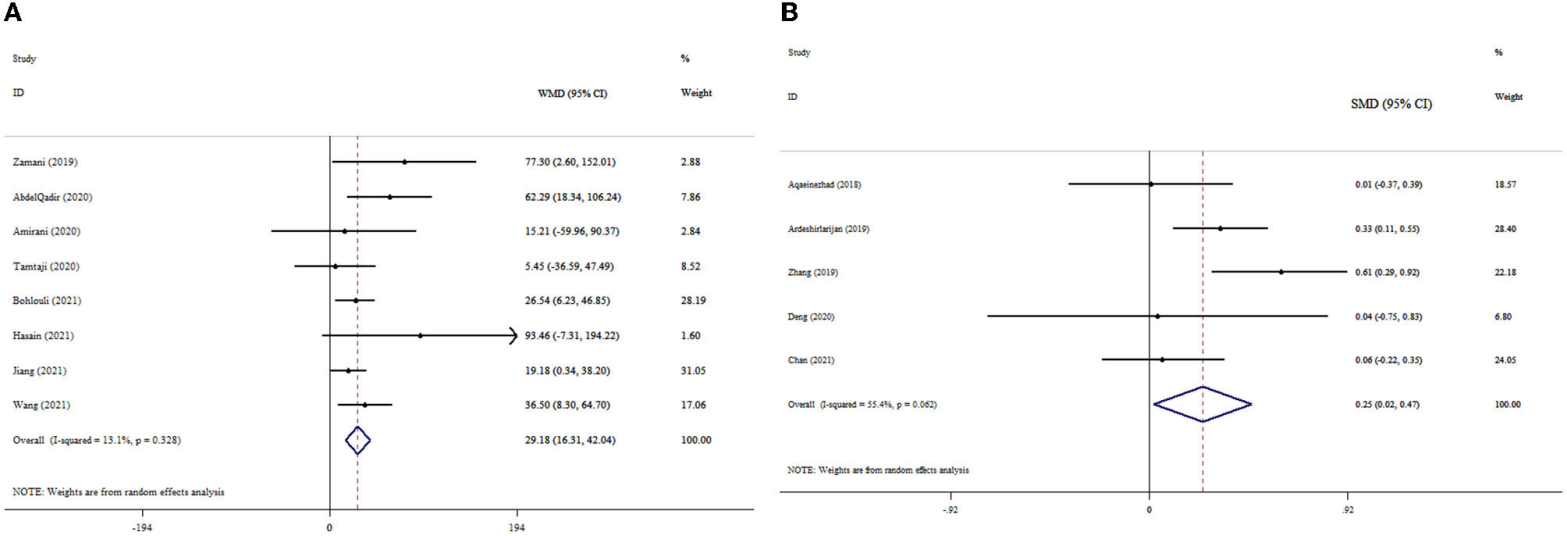
Forest plot with mean difference and 95% confidence intervals (CIs), and the effects of probiotics supplementation on TAC levels according to WMD **(A)** and SMD **(B)** analysis.

The pooled estimate of meta-analyses revealed significant improvement in the TAC levels following probiotics supplementation (ES_WMD_ = 29.18, 95% CI: 16.31, 42.04; *p* < 0.001); (I^2^ = 13.1%; *p* = 0.328) (Figure 3A). Subgroup analysis also indicated a significant impact of probiotics on TAC levels in studies with T2DM patients and a dosage of <0.4 × 10^10^ CFU (Table 3). In the sensitivity analysis, omitting each study did not substantially alter the pooled effect size for TAC. No publication bias was detected following Begg's test (*p* = 0.266).

### 3.6. The effects of probiotics supplementation on TAC based on SMD analysis

The overall analysis of the data from five studies demonstrated that probiotics supplementation significantly increased TAC levels (ES_SMD_ = 0.25, 95% CI: 0.02, 0.47; *p* = 0.032) (Figure 3B). However, no significant degree of heterogeneity existed (I^2^ = 55.4%, *p* = 0.062). The pooled effect size did not alter when each study was excluded from the sensitivity analysis. No publication bias was found by Begg's test (*p* = 0.998).

### 3.7. The effects of probiotics supplementation on GSH based on WMD analysis

**Figure 4:**
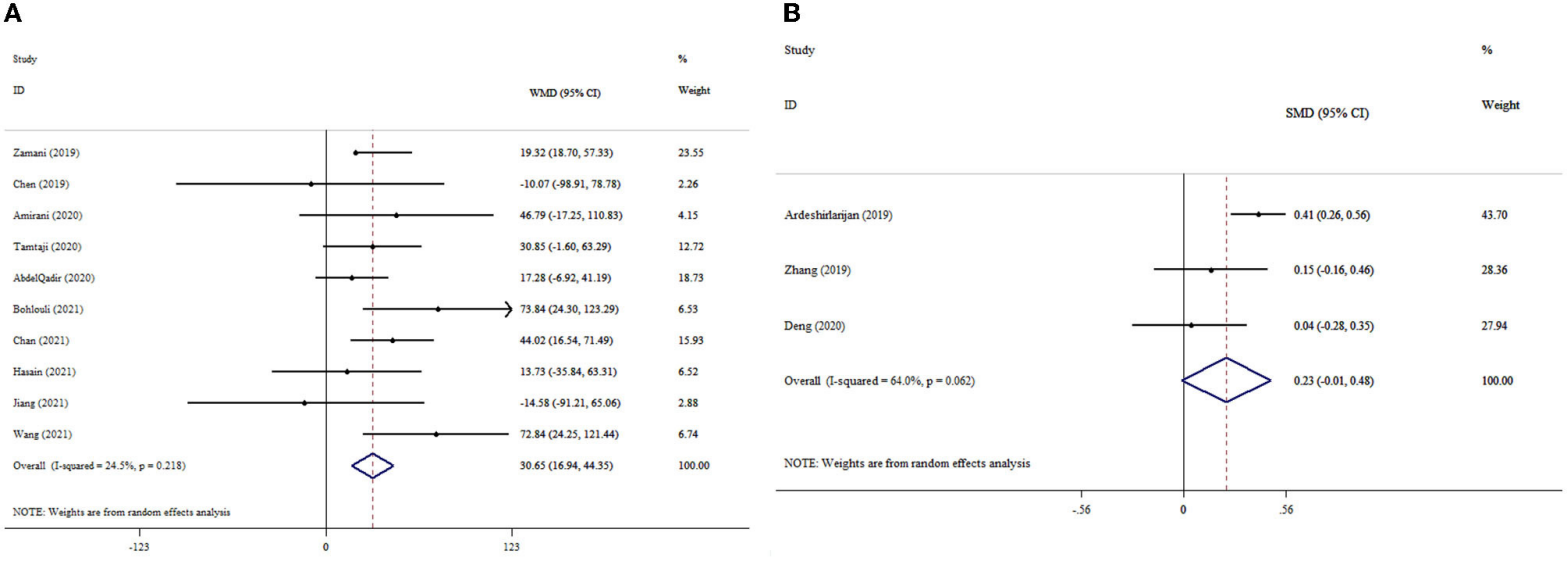
Forest plot with mean difference and 95% confidence intervals (CIs), and the effects of probiotics supplementation on GSH levels according to WMD **(A)** and SMD **(B)** analysis.

The results of the pooled analysis demonstrated that probiotics had a significant increase in GSH levels (ES_WMD_: 30.65; 95% CI: 16.94, 44.35, *p* < 0.001) (Figure 4A). There was no significant between-study heterogeneity (I^2^ = 24.5%, *p* = 0.218). Probiotics supplementation in studies with T2DM patients, a dosage of <0.4 × 10^10^ CFU, and an intervention duration of ≥10 weeks led to a remarkable increase in the GSH level compared to other subgroups (Table 3). The pooled effect size was not affected by the exclusion of any individual study using sensitivity analysis. No small-study effect was detected by Egger's and Begg's tests (*p* = 0.614 and 0.858, respectively). The funnel plot (Supplementary Figure S2) likewise did not reveal an uneven distribution of studies.

### 3.8. The effects of probiotics supplementation on GSH based on SMD analysis

Of the included studies, three studies reported the effect of probiotic supplementation on GSH levels with 1,122 participants. The results demonstrated that probiotic supplementation led to no meaningful increase in the GSH level (ES_SMD_: 0.23; 95% CI: −0.01, 0.48, *p* = 0.061) (Figure 4B). However, heterogeneity among the studies was high (I^2^ = 64.0%, *p* = 0.062). As a result of Begg's test, no publication bias was identified (*p* = 0.296).

### 3.9. The effects of probiotics supplementation on NO based on WMD analysis

**Figure 5:**
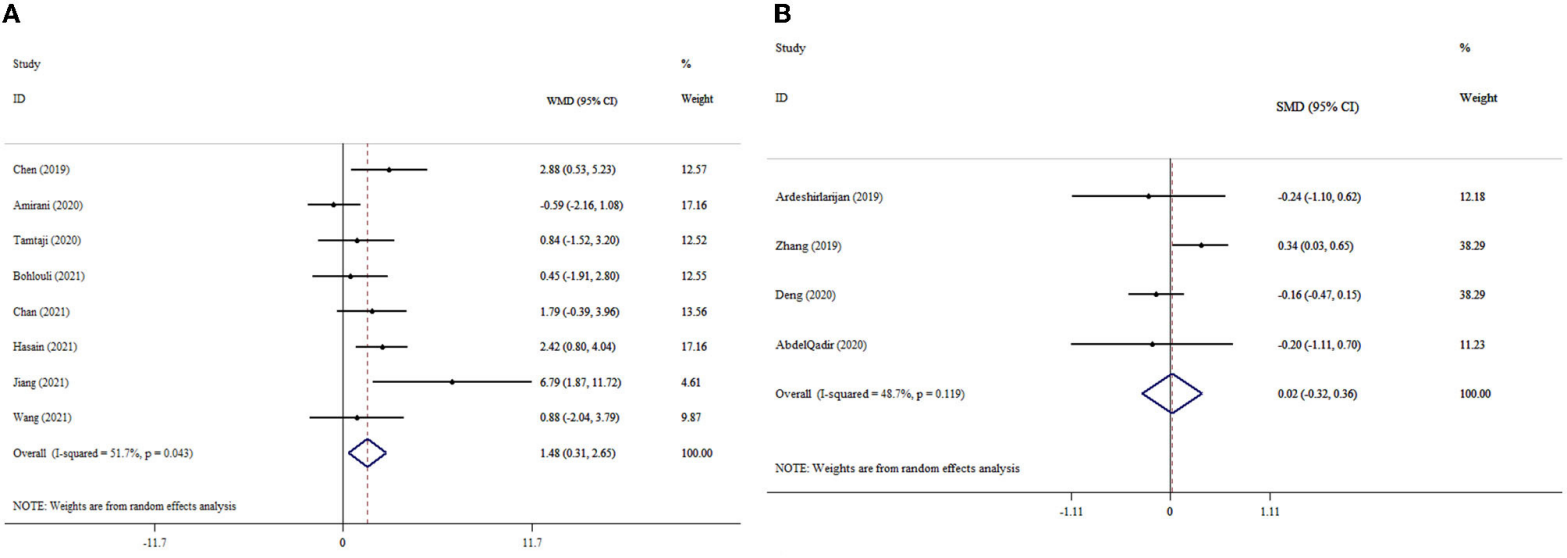
Forest plot with mean difference and 95% confidence intervals (CIs), and the effects of probiotics supplementation on NO levels according to WMD **(A)** and SMD **(B)** analysis.

The results indicated the meaningful effect of probiotics supplementation on NO levels (ES_WMD_: 1.48; 95% CI: 0.31, 2.65, *p* = 0.013; I^2^ = 51.7%, *p* = 0.043) (Figure 5A). Probiotics supplementation in the context of a dosage of <0.4 × 10^10^ CFU, mean age of <50 years, and duration of intervention of <10 weeks was greater than the overall results (Table 3). In the sensitivity analysis, any single study excluded did not impact the overall effect size. The finding of Begg's test was not significant in respect to detected publication bias (*p* = 0.618).

### 3.10. The effects of probiotics supplementation on NO based on SMD analysis

Probiotics supplementation had no significant impact on NO levels (ES_SMD_: 0.02; 95% CI: −0.32, 0.36, *p* = 0.921; I^2^ = 48.7%, *p* = 0.119) (Figure 5B). The sensitivity analysis did not influence the pooled effect size by excluding any particular study. There was no evidence of significant publication bias (*p* = 0.999 for Begg's test).

## 4. Discussion

According to our umbrella analysis, probiotics had improving effects on oxidative stress status and antioxidant biomarkers. Examining SMD and WMD showed that probiotics significantly improved antioxidant and oxidative stress biomarkers in both examinations, resulting in the ineffectiveness of standard deviation in the final result. WMD depends on the weight of each study (39). Therefore, the greater improving effect size in the WMD estimation was not surprising.

Lower dosages (<0.4 × 10^10^ CFU) of probiotics led to a maximum decrease in MDA and increase in GSH, TAC, and NO levels. Shorter (<10-week) and longer (≥10-week) durations of probiotic supplementation had the most improving effects on oxidative stress and antioxidant status, respectively. The beneficial effect of probiotics on MDA in the short term may be due to its improving effect on SOD activity in the early stages of oxidative stress. MDA is a secondary lipid peroxidation product generated by the oxidation of arachidonic acid and larger PUFAs (40). Superoxide dismutase (SOD) is the first line of defense against oxidation (41). However, due to the limited number of studies on SOD, it was not included in our analysis. Roshan et al., in a meta-analysis study, revealed that probiotics have an improving effect on SOD activity (42). GSH is involved in the next steps against oxidative stress, with the neutralization of H_2_O_2_ produced by SOD activity (41). Therefore, longer durations of probiotics are needed to affect GSH as well. TAC is not a specific measure and only presents the total status of antioxidant capacity. TAC does not evaluate SOD, glutathione peroxidase (GPx), and catalase activity (43). Theoretically, as a total antioxidant index, a longer duration of supplementation may be needed to affect TAC. However, due to the limited number of studies of short durations, performing subgroup analysis was not possible. NO acts as a double-edged sword in the mechanism of oxidative stress. A decrease in its synthesis is associated with endothelial dysfunction and subsequent thrombosis, vasospasm, vascular inflammation, and proliferation of vascular smooth muscle cells. On the other hand, increasing the production of free radicals in oxidative stress due to reaction with NO causes the production of peroxynitrite, which contributes to vascular oxidative stress (44, 45). Therefore, proper bioavailability of NO along with increasing antioxidant capacity and reducing the production of free radicals following probiotic supplementation can lead to the improvement of endothelial dysfunction and inflammation. In terms of administered dosage, a safety assessment of probiotics revealed that a low dose of probiotics may have more preventing usages than high dosages (46).

Patients with glucose intolerance have been shown to benefit more from the antioxidant effects of probiotic supplementation. One of the underlying factors in the development of diabetes mellitus is oxidative stress. Hyperglycemia through the polyol pathway, auto-oxidation, and increased production of advanced glycation end products (AGEs) contributes to the elevation of oxidative stress (47). Probiotic supplementation has also been demonstrated to exert antioxidant effects in patients with chronic kidney disease (CKD) and autoimmune diseases. The decrease of NO bioavailability and inflammation are the causes of oxidative stress in CKD patients. Moreover, the loss of antioxidant vitamins through the dialysis process and the production of ROS on the surface of dialysis membranes by the activation of neutrophils lead to oxidative stress in these patients (48). Oxidative stress through post-translational modifications of proteins is involved in the breaking of immunological tolerance and subsequent autoimmune reactions (49).

Various underlying mechanisms have been proposed for the association between probiotics and the antioxidant defense system. One is their ability to chelate metal ions. Cell-free supernatants of lactic acid bacteria (LAB) strains have been shown to exhibit the ability to chelate with metal ions such as ferrous and cupric ions (50–52). Moreover, probiotics have their own antioxidant system including SOD and catalase (53). LeBlanc et al. reported that mice with Crohn's disease receiving SOD and catalase-producing LAB had an increased antioxidant capacity in the gut (54). Probiotics also produce various metabolites related to the antioxidant system including GSH, butyrate, and B vitamins. Folate is important in the efficiency of DNA replication, repair, and methylation (55). Numerous studies have reported that various probiotic strains including LAB and *Bifidobacteria* are able to produce folate and enhance its status in human and animal models (56–58). *B. longum* and *B. bifidum* have been shown to be able to produce thiamin (59); moreover, *L. fermentum* can produce riboflavin (60). Furthermore, the ability of some strains in the production of vitamin B12 has been shown in studies (61–63). Elevation of the homocysteine level as the main consequence of B-vitamins deficiency is the cause of oxidative stress in this condition (64). Results on fat-soluble vitamins are limited, and an exact conclusion cannot be obtained (65). Kullisaar et al. reported that *Lactobacillus fermentum* E-3 and E-18 had significant levels of GSH (66). In addition, a whole GSH system was found in *Lactobacillus fermentum* ME-3 (67). The beneficial effect of sodium butyrate on the oxidative status through the partial activation of nuclear factor-erythroid factor 2-related factor 2 (Nrf2)-dependent genes has been reported in an *in vivo* investigation (68). Butyrate is a short-chain fatty acid (SCFA) whose production by *Clostridium butyricum* was studied in an *in vivo* model (69). In addition, probiotics can regulate signaling pathways related to antioxidant responses including the induction of Nrf2-Keap1-ARE (70, 71), mitogen-activated protein kinases (MAPKs) (72), and protein kinase C (PKC) (72), as well as the inhibition of nuclear factor kappa-B (NFκB) (73). Kelch-like ECH-associated protein 1 (Keap-1) is a molecular switcher that activates Nrf2 when cells are counteracted with free radicals. Subsequently, Nrf2 increases the expression of antioxidant enzymes and detoxifying proteins by binding to the antioxidant response element (ARE) (74). NFκB is the main transcription factor involved in the regulation of inflammatory pathways (75). Both MAPKs and PKC are the enzymes involved in the signaling pathways leading to the regulation of cell growth. Probiotic-secretory proteins through the PKC- and MAPK-dependent mechanism protect intestinal integrity (72). Another possible antioxidant aspect of probiotics is their inhibitory effect on enzymes producing free radicals, including the NADPH oxidase (NOX) complex (76), cyclo-oxygenase (COX) (77), and cytochrome P450 (CYP) enzymes (78).

In terms of bias, 11 of included studies reported that the majority of their analyzed clinical trials had a low risk of bias. Moreover, 5 and 8 of the 15 included systematic review studies had a high and moderate risk of bias, respectively. Therefore, our obtained results can be almost reliable. However, there are some limitations to our study. First, due to the limited number of studies, subgroup analysis based on study duration on TAC was not performed. Second, due to the presence of a wide range of health conditions, subgroup analysis based on diseases other than diabetes mellitus was not possible. This problem also applies to different strains of probiotics.

## 5. Conclusion

Probiotics in low dosages can be considered as antioxidant agents. Shorter (<10-week) and longer (≥10-week) durations of probiotic supplementation have the most improving effects on oxidative stress and antioxidant status, respectively. Patients with different health conditions such as T2DM, CKD, and autoimmune diseases can benefit from probiotic supplementation.

## Data availability statement

The original contributions presented in the study are included in the article/Supplementary material, further inquiries can be directed to the corresponding authors.

## Author contributions

VM and MZ designed research. AF and VM conducted the systematic search. MZ, AP, and FH-K screened the articles. FH-K, AJ, and PM extracted the data. MZ analyzed and interpreted the data. AJ and FH-K drew the tables. MZ, VM, and AF wrote the paper. ZG had primary responsibility for the final content. All authors read and approved the final manuscript.

## Supplementary material

Supplementary Figures The results of funnel plot for the effect of probiotics on oxidative stress.
