# Corrigendum: Remarkable impacts of probiotics supplementation in enhancing of the antioxidant status: results of an umbrella meta-analysis

**DOI:** 10.3389/fnut.2024.1371746

**Published:** 2024-02-01

**Authors:** Vali Musazadeh, Amir Hossein Faghfouri, Meysam Zarezadeh, Azin Pakmehr, Pooria Taghavi Moghaddam, Fateme Hamedi-Kalajahi, Arian Jahandideh, Zohreh Ghoreishi

**Affiliations:** ^1^Student Research Committee, Tabriz University of Medical Sciences, Tabriz, Iran; ^2^School of Nutrition and Food Sciences, Tabriz University of Medical Sciences, Tabriz, Iran; ^3^Maternal and Childhood Obesity Research Center, Urmia University of Medical Sciences, Urmia, Iran; ^4^Nutrition Research Center, Faculty of Nutrition and Food Science, Tabriz University of Medical Sciences, Tabriz, Iran; ^5^Faculty of Medicine, Tehran University of Medical Sciences, Tehran, Iran; ^6^Department of Pharmaceutics, School of Pharmacy, Ahvaz Jundishapur University of Medical Sciences, Ahvaz, Iran; ^7^Usern Office, Mazandaran University of Medical Sciences, Sari, Iran

**Keywords:** systematic review, probiotics, umbrella meta-analysis, malondialdehyde (MDA), oxidative stress biomarkers

In the published article, there was an error in affiliation for Zohreh Ghoreishi. Instead of “Affiliation 3”, it should be “Affiliation 4”.

The authors apologize for this error and state that this does not change the scientific conclusions of the article in any way. The original article has been updated.

