# Supplementary material for: Remarkable impacts of probiotics supplementation in enhancing of the antioxidant status: results of an umbrella meta-analysis
Source: Front Nutr. 2023 Aug 11;10:1117387. doi: 10.3389/fnut.2023.1117387 (PMC10451070; doi:10.3389/fnut.2023.1117387)
Supplement: Supplementary Figures — The results of funnel plot for the effect of probiotics on oxidative stress. [file Image_1.pdf]

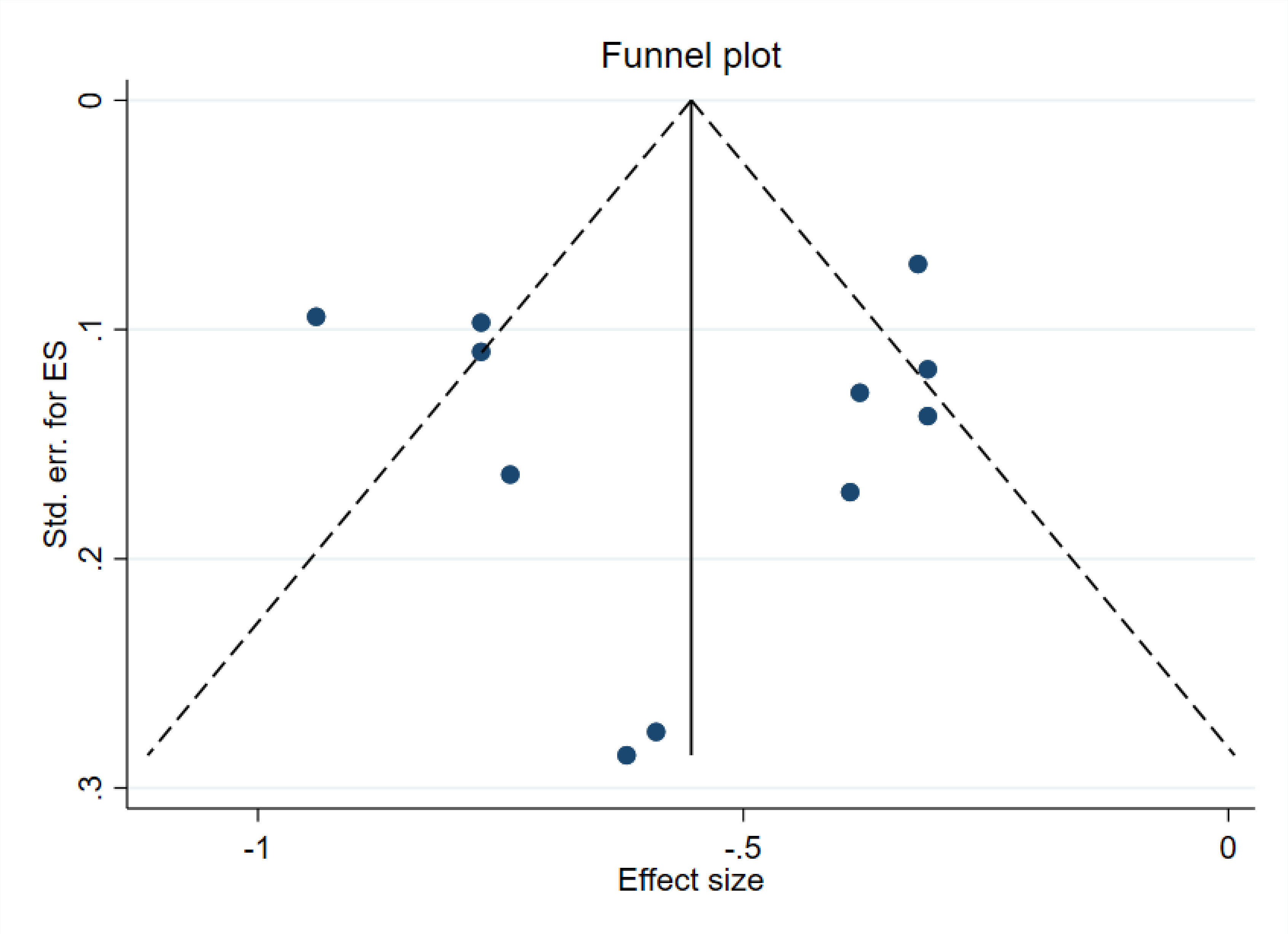

Figure S1. Funnel plot of the effect of probiotics on MDA.

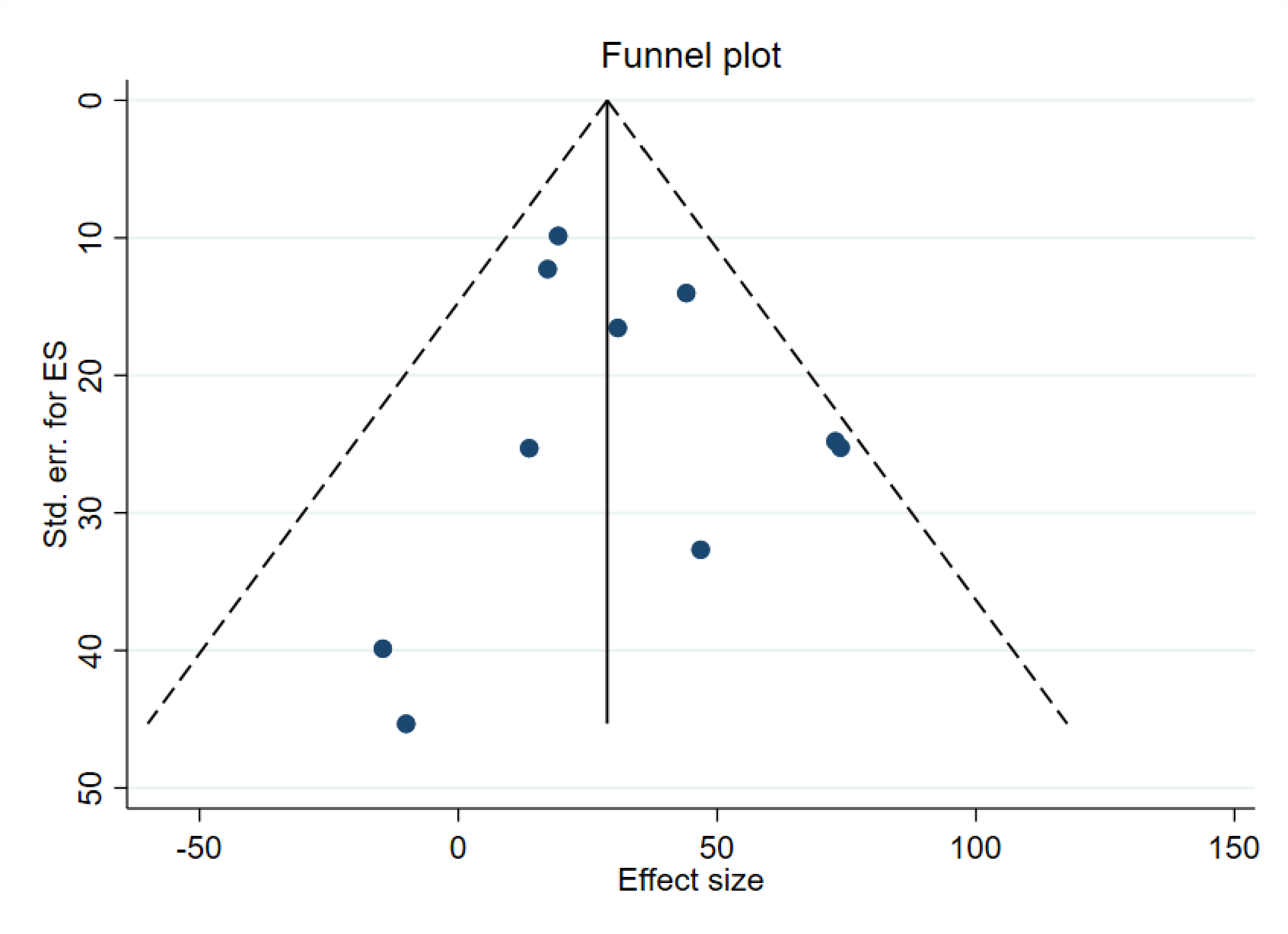

Figure S2. Funnel plot of the effect of probiotics on GSH.
